# Bone morphogenetic proteins 2, 6, and 9 differentially regulate the osteogenic differentiation of immortalized preodontoblasts

**DOI:** 10.1590/1414-431X20209750

**Published:** 2020-08-03

**Authors:** Yuying Cao, Qin Tan, Jing Li, Jinhua Wang

**Affiliations:** 1 Stomatological Hospital of Chongqing Medical University, Chongqing, China; 2 Chongqing Key Laboratory of Oral Diseases and Biomedical Sciences, The Affiliated Hospital of Stomatology of Chongqing Medical University, Chongqing, China; 3 Chongqing Municipal Key Laboratory of Oral Biomedical Engineering of Higher Education, College of Stomatology, Chongqing Medical University, Chongqing, China

**Keywords:** Preodontoblast, Immortalized preodontoblast, Bone morphogenetic proteins, BMP2/6/9, Osteogenic differentiation

## Abstract

Our study attempted to compare the efficacies of bone morphogenetic protein (BMP) 2, 6, and 9 in inducing osteogenic differentiation of preodontoblasts (PDBs). We immortalized PDBs by introducing a reversible SV40 T antigen-based immortalization system. Cell proliferation capability was examined by the 3-(4,5-dimethyl-2-thiazolyl)-2,5-diphenyl-2-H-tetrazolium bromide assay. The effects of BMP2, 6, and 9 on the osteogenic differentiation of immortalized preodontoblasts (iPDBs) were measured by alkaline phosphatase (ALP) activity assays and alizarin red S staining. The expression of osteogenic markers was evaluated by semiquantitative real-time polymerase chain reaction analysis. To assess ectopic bone formation, rat-derived iPDBs were transfected in culture with adenoviral vectors designated Ad-BMP2, 6, and 9 and subcutaneously or intramuscularly injected into mice. Several BMPs retained endogenous expression in PDBs and regulated the mRNA expression of mineralized tissue-associated proteins. ALP activity and mineralized nodule formation were significantly increased in the Ad-BMP9-transfected group relative to the control group. In addition, the most significant hard tissue formation was in this group. The results indicated that BMP signaling was involved in the osteogenic differentiation of iPDBs. BMP9 could be an efficacious accelerant of the osteogenic differentiation of iPDBs.

## Introduction

Bone tissue regeneration is a current challenge in the treatment of hard tissue defects. The key components of bone tissue engineering are osteogenic growth factors, stem cells, and extracellular matrix scaffolds (1). Currently, our research group is developing a rational method based on morphogenetic signals for bone tissue induction, stem/progenitor cell responses, and scaffolds to repair bone defects. Several types of mesenchymal stem cells (MSCs) are present in bone tissue, such as bone marrow MSCs, which have been used as seed cells in bone tissue engineering. Dental tissue-originated MSCs stand out as a stem cell source with broad potential for bone regeneration in various bone surgeries due to their convenient extraction, abundance, and easy isolation and purification (2). Preodontoblasts (PDBs), the progenitors of odontoblasts, derive from dental papilla and have similar bone regeneration and restoration abilities, including cell proliferation and extracellular matrix maturation and mineralization (3). Therefore, we propose that exogenous bone morphogenetic proteins (BMPs) might have the capacity to induce bone tissue-associated gene mRNA expression and mineralization of human PDBs (4,5).

Previous studies have demonstrated that in hard tissue repair, MSCs are regulated by a variety of signaling pathways, among which the BMP signaling pathway is the most widely studied pathway to date with respect to bone formation (6,7). Multifunctional differentiation factors are expressed in limb growth, endochondral ossification, early fracture, and cartilage repair and have essential roles in embryonic development and the regeneration of bone and cartilage, which are pivotal to MSC osteogenic differentiation (8). BMPs can stimulate DNA synthesis and cell proliferation, thereby promoting the differentiation of MSCs into osteoblasts. Stem/progenitor cells in dental pulp tissue can potentially differentiate into osteoblasts and participate in bone matrix secretion and mineralization in response to BMPs. Previous studies reported that, among a dozen BMPs, BMP2, 6, and 9 were the most osteogenic and that BMP9 induced the most potent osteogenic differentiation activity among the members of the BMP family (9,10). In the present study, we investigated the osteogenesis of PDBs induced by BMP2, 6, and 9.

## Material and Methods

### Cell culture and immortalization of rat immortalized PDBs (iPDBs)

Early-passage (third-passage) PDBs were infected with packaged retrovirus SSR#69, which expresses SV40 T antigen flanked with Cre/loxP sites. Stable iPDB lines were established by selecting the infected cells with hygromycin B for 1 week (3,10–12). The iPDBs and HEK-293 cells were gifted by Molecular Oncology Laboratory, University of Chicago Medical Center (USA). Cells were cultured in complete Dulbecco's modified Eagle's medium (DMEM) supplemented with 10% (v/v) fetal bovine serum (FBS), 100 U/mL penicillin, and 100 µg/mL streptomycin at 37°C in a humidified atmosphere of 5% CO_2_. All chemicals were purchased from Fisher Scientific (USA) or Sigma-Aldrich (USA) unless otherwise specified.

### Transfection of recombinant adenoviruses expressing BMP2, 6, or 9 and green fluorescent protein (GFP)

Recombinant adenovirus generation and transfection efficiency have been reported previously (13,14). The coding regions for human BMP2, 6, and 9 and Cre recombinase were PCR amplified and subcloned into a shuttle vector, which was used to generate recombinant adenoviruses in HEK-293 cells by Ad-Easy technology (Molecular Oncology Laboratory, University of Chicago Medical Center). The resulting adenoviral vectors were designated as Ad-BMP2, 6, and 9, and an analogous adenoviral vector was used to express only GFP (Ad-GFP) as a control. Polybrene (10 mg/mL) was used to enhance transduction efficiency for adenoviral infection. At 48 h after infection, the GFP signals of infected iPDBs were observed by inverted fluorescence microscopy (Carl Zeiss, Germany).

### MTT proliferation assays

Cell proliferative activity was determined using the 3-(4,5-dimethyl-2-thiazolyl)-2,5-diphenyl-2-H-tetrazolium bromide (MTT) method (Beyotime, China) (15,16). iPDBs and PDBs were seeded in 96-well plates in 100 µL of growth medium and cultured for another 1, 2, 3, 4, 5, 6, and 7 days. Subsequently, 15 µL of MTT solution (5 mg/mL) was added to each well. After 4-h incubation, cells were disrupted in 100 µL of dimethyl sulfoxide (DMSO), and absorbance at 570 nm was measured on a microplate reader (BioTek, USA).

### RNA isolation and semiquantitative real-time polymerase chain reaction (RT-PCR)

For semiquantitative RT-PCR, total RNA was extracted using TRIzol Reagent (Invitrogen, USA) according to the manufacturer's instructions and used to synthesize cDNA templates using reverse transcription reactions with hexamer and M-MuLV reverse transcriptase (New England Biolabs, USA). The first-strand cDNA products were diluted 5–10-fold and used as PCR templates. SYBR Green-based RT-PCR analysis was carried out using the thermocycler Opticon II DNA Engine (Bio-Rad, USA) with a standard pUC19 plasmid (3,11,17,18). The markers bone sialoprotein (BSP), matrix extracellular phosphoglycoprotein (MEPE), SMAD6, and dentin matrix protein 1 (DMP1) and the late osteogenic differentiation markers osteocalcin (OCN) and osteopontin (OPN) were analyzed, and GAPDH was used as the ‘housekeeping' gene to normalize RNA expression. The RT-PCR primers (Table 1) were designed using the Primer3 system (Takara, Japan).

**Table 1:** Primer sequences for specific genes associated with osteogenic differentiation.

Gene	Primer sequence (5′-3′)
GAPDH	5′AAGCTGTGGACGCTTTGG3′
	5′ATCCAGGGAGCGAGGAAT3′
BSP	5′AAAGTGAAGGAAAGCGACGA3′
	5′CGGCCTTCTGCACCTGCTTC3′
DMP1	5′GGAACTGCAGCACAGAATGA3′
	5′CAGTGTTCCCCTGTTCGTTT3′
MEPE	5′ACAAGTGGCCTCGAGAGAAA3′
	5′CTGGCTTTTGCCTTTACCTG3′
OCN	5′CTGACCTCACAGATCCCAAGC3′
	5′TGGTCTGATAGCTCGTCACAA3′
OPN	5′CACTCCAATCGTCCCTACAGT3′
	5′CTGGAAACTCCTAGACTTTGAC3′
SMAD6	5′CGGGTTACTCCATCAAGGTGTT3′
	5′CAGGAGGTGATGAACTGTCGC3′

### Alkaline phosphatase (ALP) activity assay

ALP activity is a typical marker of early osteoblastic differentiation. To examine whether the upregulation of BMP2, 6, or 9 induced ALP activity in iPDBs, cells were divided into four groups before being transfected with recombinant adenoviruses BMP2, 6, or 9 or GFP for 3, 5, 7, or 9 days. ALP activity was determined quantitatively with a modified Great Escape SEAP chemiluminescence assay (BD, Clontech, USA) and/or histochemical staining, as described previously (9,19).

### Alizarin red S (ARS) staining

iPDBs were seeded in 24-well cell culture plates and infected with Ad-BMP2, 6, 9, or Ad-GFP. Infected cells were cultured in the presence of ascorbic acid (50 mg/mL) and β-glycerophosphate (10 mM). At 14 days after infection, the cells were stained with ARS to examine mineralized nodule formation and calcium deposition as described previously (11,19). Briefly, cells were fixed with 0.05% (v/v) glutaraldehyde at room temperature. After 10 min, cells were incubated in 0.4% ARS (Sigma-Aldrich Corporation, USA) at a pH of 4.0 for 15 min at room temperature. Cells were then extensively washed with distilled water. The staining of calcium mineral nodules was recorded under bright field microscopy.

### Cell implantation and ectopic bone tissue formation

The animal studies were conducted in accordance with the guidelines approved by the Ethics Committee of Chongqing Medicine University (China). Stem cell-mediated ectopic bone formation was performed as described previously (3,10,11). Briefly, cells were infected with Ad-BMP2, 6, 9, or Ad-GFP for 24 h and then collected and resuspended in PBS for subcutaneous or intramuscular injection (1×10^6^ cells/injection) into the flanks of athymic (nu/nu) nude mice (5 animals per group, 4-6 weeks old, female, Experimental Animal Center of Chongqing Medicine University, China). Six weeks after injection, the animals were sacrificed and samples were retrieved. The implantation sites were examined for microcomputed tomography (μ-CT) imaging, histological evaluation, and staining.

### Microcomputed tomography analysis

The retrieved specimens from the implantation sites were fixed and measured with the μ-CT function on a Viva CT 40 scanner (Scanco Medical, Switzerland) at 70 kV voltage with a 114 µA beam current. The image data analysis was performed using µ-CT V6.1 software (Scanco Medical) to evaluate bone volume as described previously (3,20).

### Histological evaluation

Retrieved bone masses and cultured tissues were harvested and fixed in a neutral paraformaldehyde solution, demineralized with 10% ethylene diamine tetra-acetic acid (EDTA), and embedded in paraffin. Tissue sections of the embedded specimens were stained with hematoxylin and eosin (H&E) for morphogenetic study as described before (11,20,21). Masson's trichrome staining and alcian blue staining were also carried out after the sections were deparaffinized and rehydrated.

### Statistical analysis

For all quantitative assays, each assay condition was performed in triplicate. Microsoft Excel was used to calculate the standard deviations (SD) and identify statistically significant differences among samples by one-way analysis of variance (ANOVA) and Scheffe's multiple comparison test. A significant difference was defined as a P-value less than 0.05.

## Results

### Cell proliferation and BMP endogenous expression in iPDBs

**Figure 1:**
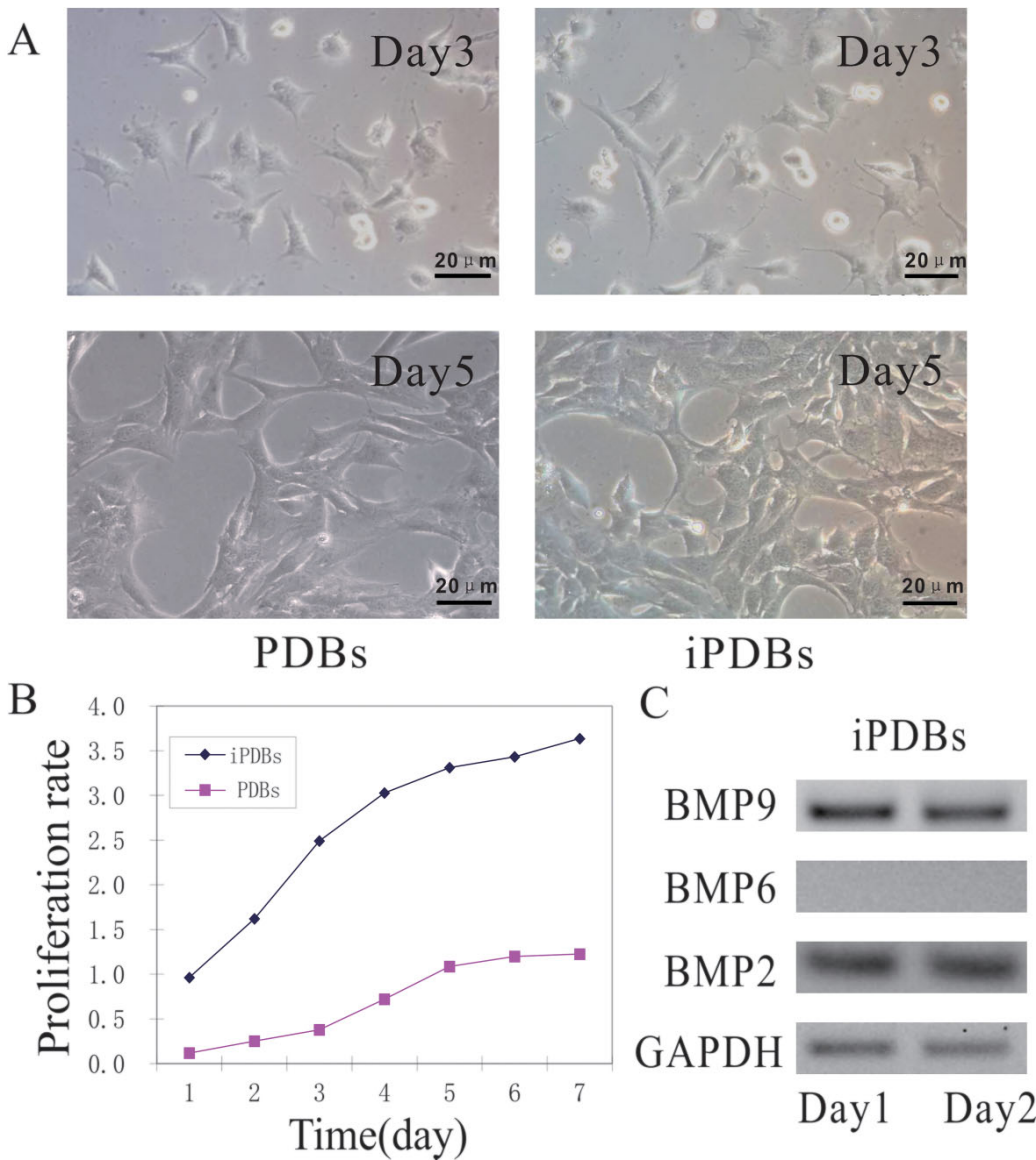
Cell morphology, proliferation activity, and bone morphogenetic proteins (BMPs) endogenous expression. The preodontoblasts (PDBs) and immortalized preodontoblasts (iPDBs) were seeded at a low confluence (**A**) and subjected to MTT assays at the indicated time points (scale bars 20 μm). The iPDBs had a higher growth rate than PDBs (**B**). The endogenous expressions of BMP2, 6, and 9 were measured using semi-quantitative RT-PCR assay in iPDBs (**C**). Each assay was done in triplicate.

The iPDBs showed a higher cell proliferation rate than the PDBs at each indicated time point (Figure 1A and B). To confirm that iPDBs express BMP-related genes, we measured the endogenous expression of BMP2, 6, and 9 using an RT-PCR assay. BMP9 expression was pronounced, while BMP6 expression was almost undetectable in iPDBs. The expression of BMPs on day 2 was slightly higher than that on day 1 (Figure 1C).

### iPDBs differentiated into osteogenic lineages upon BMP2, 6, or 9 stimulation

**Figure 2:**
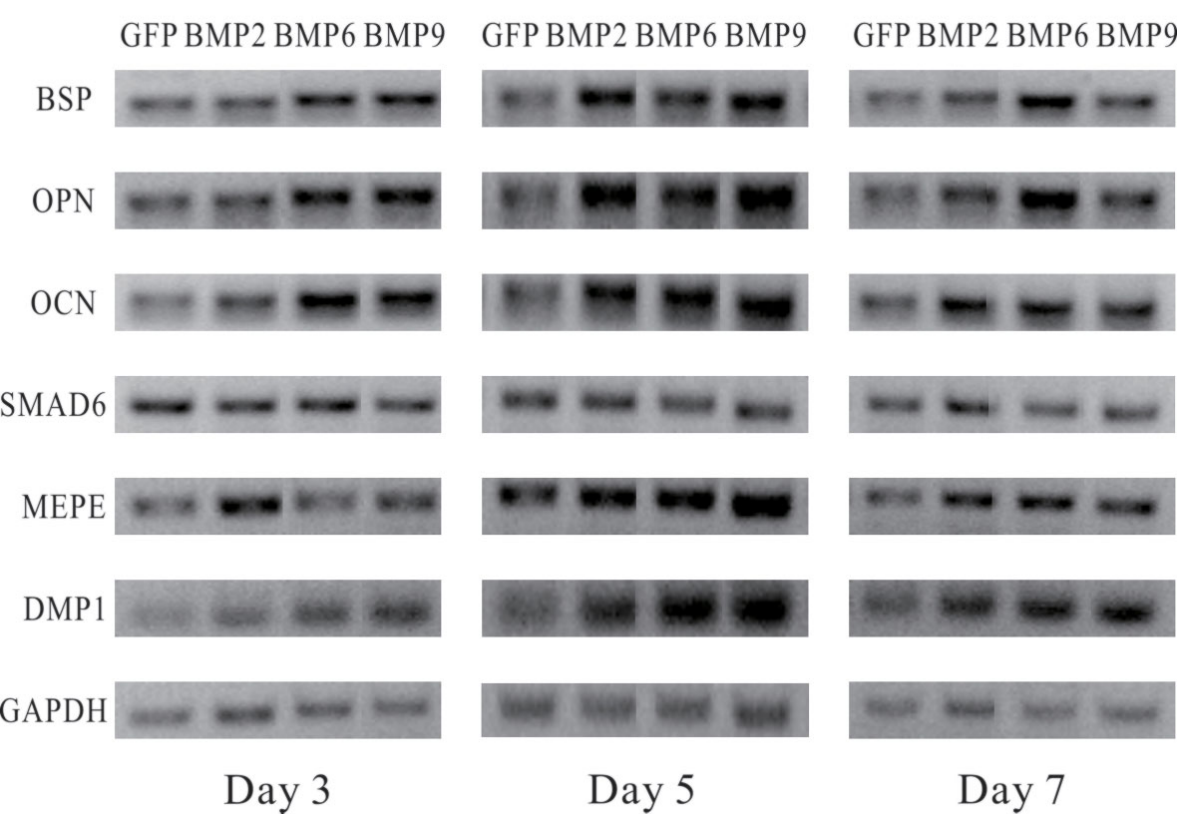
Representative results of semi-quantitative RT-PCR analysis of osteogenesis-related genes in immortalized preodontoblasts (iPDBs) upon bone morphogenetic proteins (BMPs) stimulation. Relative expression level was calculated and standardized with endogenous GAPDH expression levels. Each assay was done in triplicate. GFP: green fluorescent protein; BMP: bone morphogenetic protein; BSP: bone sialoprotein; OPN: osteopontin; OCN: osteocalcin; MEPE: matrix extracellular phosphoglycoprotein; DMP1: dentin matrix protein 1.

As odontoblasts can express MSC markers (9,22), we tested whether iPDBs are able to differentiate into osteogenic lineages. The expression of osteogenic markers was upregulated with higher endogenous expression. BSP, OPN, and OCN expression were significantly upregulated after induction with BMP2, 6, or 9 as early as day 3, while DMP1 and MEPE expression were upregulated, achieving higher levels at later time points. In contrast, SMAD6, a kind of osteogenic differentiation antagonist gene that blocks BMP signaling pathways, showed no obvious difference in expression between the BMP-induced and GFP groups, but its expression was lower in the BMP9-treated group than in the GFP group (Figure 2).

Overall, iPDBs increased the osteogenic potential after BMP induction; in addition, the expression levels of osteogenesis-related genes were upregulated. These results strongly suggested that BMP signaling may participate in proliferative expansion of progenitors and eventual osteogenic terminal differentiation. BMP signaling played an essential role in the osteogenic differentiation of iPDBs.

### BMPs upregulated ALP activity and mineralized nodules

**Figure 3:**
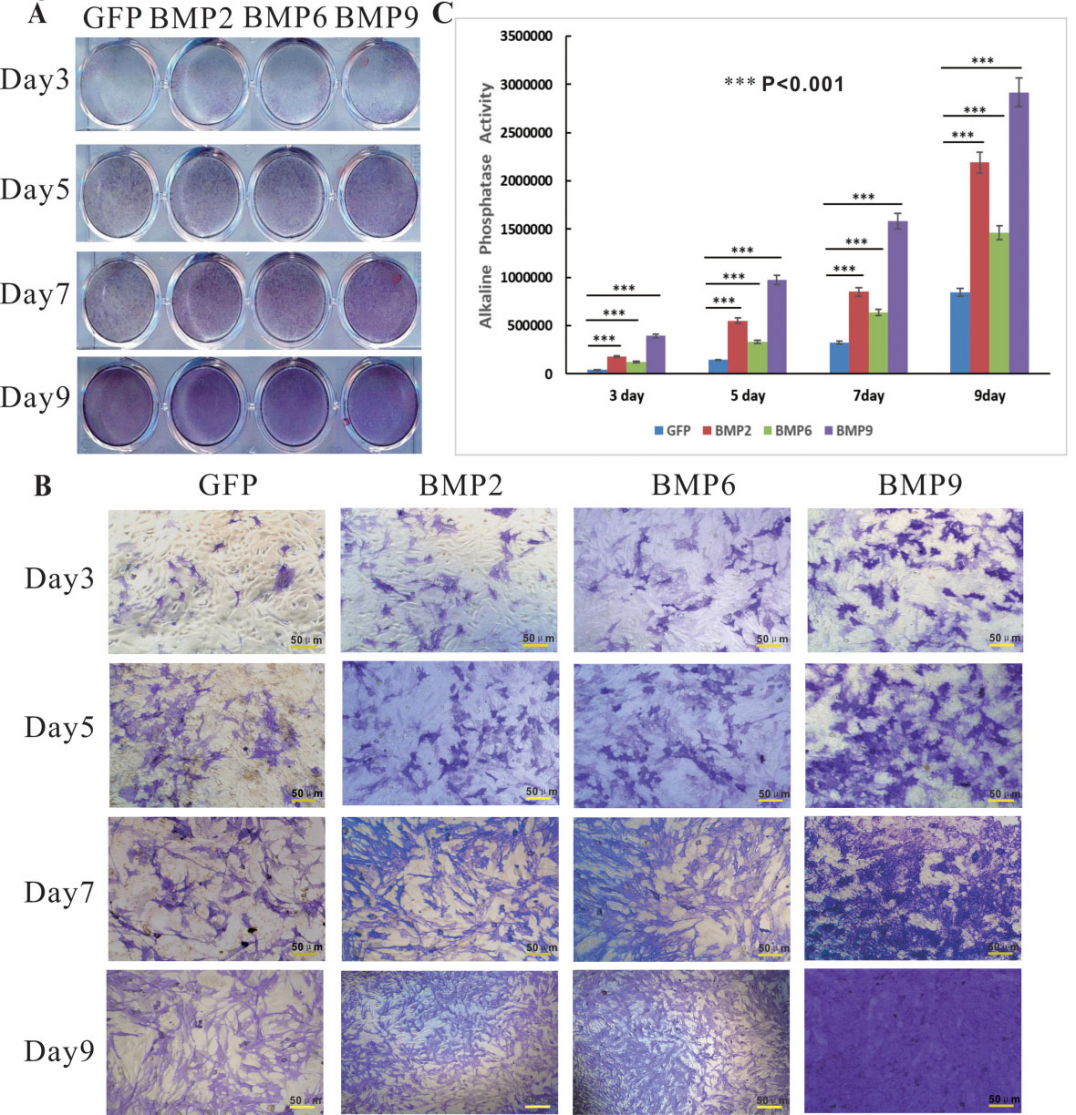
Bone morphogenetic proteins (BMPs) promote early mineralized marker alkaline phosphatase (ALP) activity. Subconfluent cells were seeded in 24-well cell culture plates and infected with Ad-BMP2, 6, 9, or Ad-GFP. At the indicated time points post-infection, cells were fixed and histochemically stained for ALP activity (**A** and **B;** scale bars 50 μm) or subjected to ALP quantitative analysis at the indicated time-points (**C**). Data are reported as means±SD. Each assay was performed in triplicate. ***P<0.001 (ANOVA). GFP: green fluorescent protein.

As a marker of early mineralization, we sought to examine whether BMP2, 6, and 9 can induce the activity of the osteogenic marker ALP in iPDBs. When cells were induced in calcifying medium, ALP activity was readily detected in the cells stimulated with BMP9, while lower ALP activity was detected in the BMP2- and 6-treated groups; however, ALP activity gradually increased over time (Figure 3A). There were no significant differences in ALP activity between the BMP2- and 6-induced groups and the GFP control group after 3, 5, and 7 days of induction. On day 9, ALP activity in the BMP2-induced group was much higher than that in the BMP6-induced group (Figure 3B). Quantitative analysis of ALP activity in iPDBs revealed similar results across multiple time points (Figure 3C) (P<0.001).

**Figure 4:**
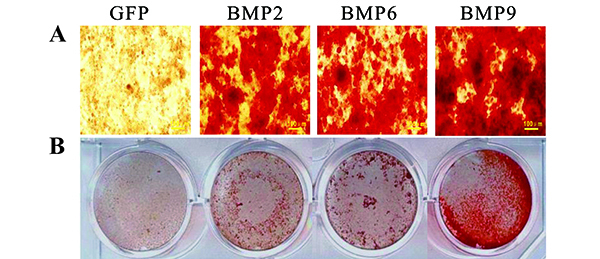
Bone morphogenetic proteins (BMPs) promote late-stage mineral deposition in immortalized preodontoblasts (iPDBs). Ad-BMP2, 6, 9, or Ad-GFP infected iPDBs were seeded in 24-well cell culture plates and then maintained in mineralization culture medium for 14 days and stained with alizarin red S (**A** and **B**). Representative images are shown (scale bars 100 μm). GFP: green fluorescent protein.

Calcified nodule formation is an important feature of late stage mineralization. To determine how BMP2, 6, and 9 affect the formation of calcified nodules in iPDBs, nodule formation assays were carried out. Mineralized nodule densities and sizes were increased with long cell induction in all groups (Figure 4A). Mineralized nodules were not detected in the GFP group, and the ARS staining results were consistent with the ALP findings. Overexpression of BMP9 resulted in significantly stronger matrix mineralization, with stronger ARS staining, than did overexpression of BMP2 or 6 (Figure 4B). These results strongly suggested that the activation of BMP signaling, especially BMP9 signaling, may facilitate the osteogenic differentiation of iPDBs.

### BMPs induced the mineralization of ectopic tissue in iPDBs

**Figure 5:**
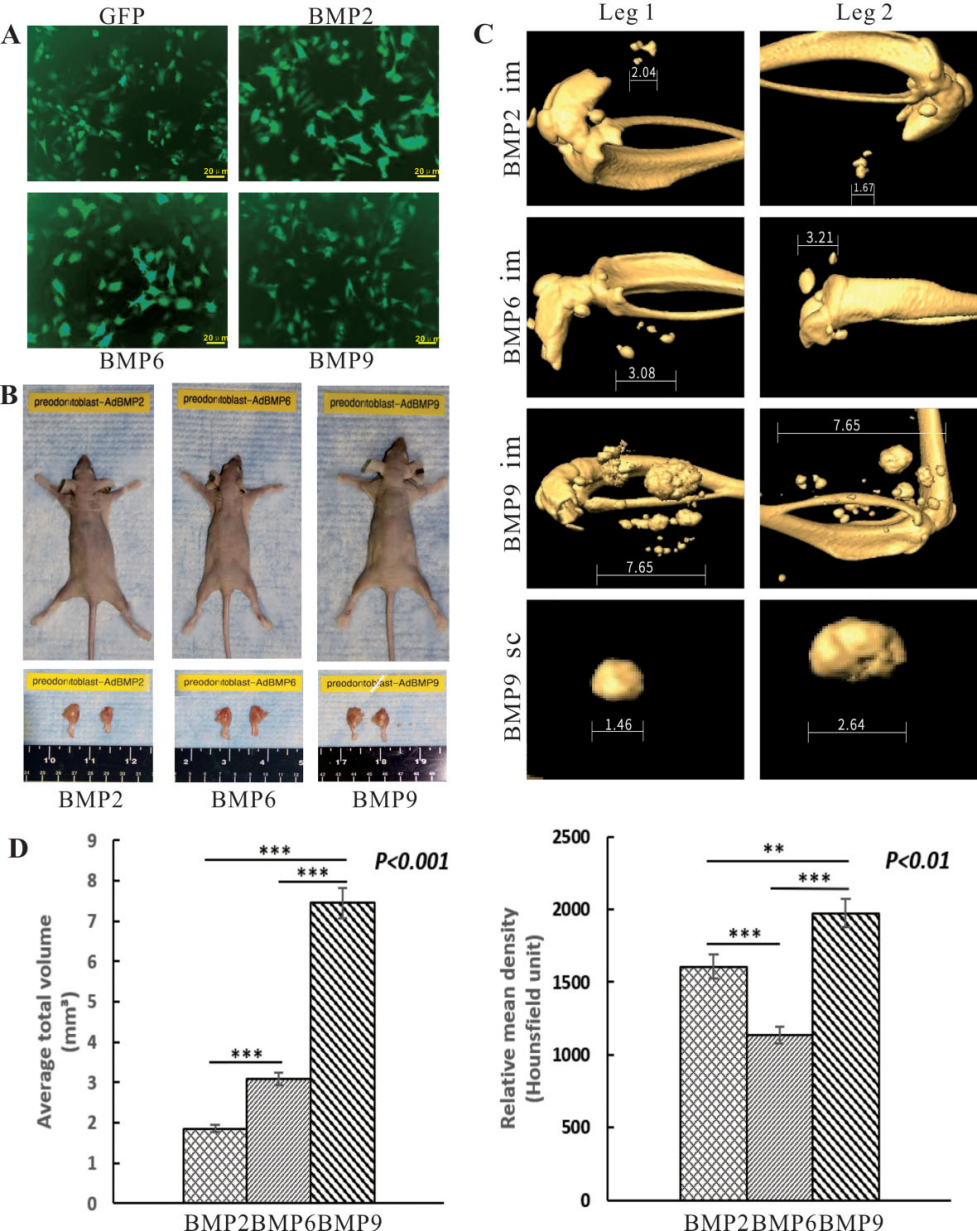
Bone morphogenetic proteins (BMPs) effectively induced osteogenesis of immortalized preodontoblasts (iPDBs) *in vivo*. Subconfluent iPDBs were equally infected with Ad-BMP2, 6, 9, or Ad-GFP (**A**; scale bars 20 μm), collected and resuspended in PBS for subcutaneous (sc) injections into the flanks, or intramuscular (im) injections [10^6^ cells/site in 100 mL PBS] of athymic nude mice. At 6 weeks after implantation, masses formed at the injection sites were retrieved (**B**), fixed in formalin, and subjected to μ-CT imaging. No detectable masses were formed in the Ad-GFP infected and subcutaneous BMP2 and 6 infected iPDBs groups. Representative 3-D isosurface reconstruction images of the retrieved masses are shown (**C**). The mean (SD) volume and density of masses were quantitatively analyzed (**D**). **P<0.01, ***P<0.001 (ANOVA). GFP: green fluorescent protein.

Using our previously established stem cell implantation assay (10,11), we further examined the bone-forming capability of iPDBs upon BMP stimulation. When equally transduced with Ad-BMP2, 6, and 9 (Figure 5A), iPDBs were collected and injected into athymic nude mice. Mineralized tissue masses were retrieved from mice injected with BMP2, 6 or 9-transduced iPDBs after 6 weeks. Only a few masses were detected in iPDBs transduced with Ad-GFP (data not shown) (Figure 5B).

μ-CT imaging indicated that iPDBs stimulated by BMPs formed substantially larger and more robust masses in the intramuscular injection group than in the subcutaneous injection group, possibly in part due to the abundant blood supply. Therefore, we mainly analyzed the retrieved masses from legs. The mineralized tissue masses of the BMP9 group were greater than those of the other groups (Figure 5C). The average volume of the mineralized masses in the BMP6 group was larger than that in the BMP2 group (P<0.001), while the mean density of the masses retrieved from the BMP6 treatment group was slightly lower than that from the BMP2 group (Figure 5D) (P<0.01).

**Figure 6:**
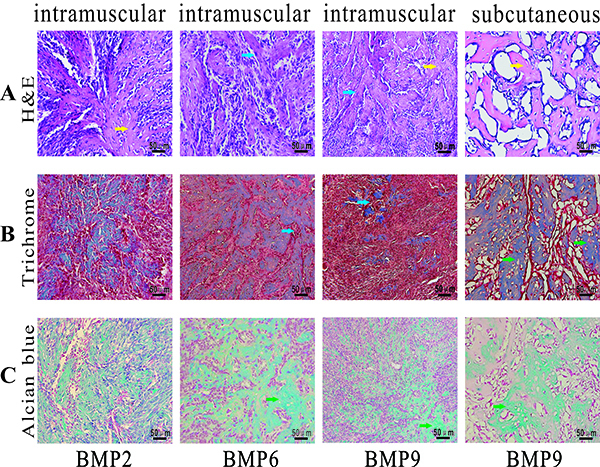
Sections of the bone morphogenetic proteins (BMPs)-treated samples were retrieved, fixed, decalcified, and subjected to paraffin-embedded sectioning for histologic evaluation. Bone-like trabeculae (blue arrows) and osteoid matrix (yellow arrows) were observed by hematoxylin and eosin (H&E) staining (**A**). Mineralized osteoid matrix (blue arrows) and cartilage-like tissues (green arrows) were observed by trichrome staining (**B**). Cartilage-like deposits were observed by alcian blue staining (**C**). Scale bars 50 μm.

H&E staining of the retrieved masses from the intramuscular injection sites indicated that the BMP9-transduced iPDBs formed more mature osteoid matrices and orderly bone-like trabeculae than did the BMP2- and 6-transduced iPDBs (Figure 6A). Trichrome staining confirmed this result, showing that BMP2 and 6 induced fewer mineralized osteoid matrices and the formation of more cartilage-like tissues *in vivo*. However, both H&E and trichrome staining indicated that the BMP2-transduced group formed more bone-like trabeculae and osteoid matrices than the BMP6-transduced group (Figure 6B). Furthermore, the presence of cartilage-like deposits was found in all groups as evidenced by alcian blue staining, but BMP9-transduced iPDBs formed fewer cartilage-like deposits compared with the other groups (Figure 6C). Collectively, the ectopic results strongly supported the common view that iPDBs transfected with BMP9 can induce the formation of mature and orderly bone-like trabeculae and cartilage-like deposition.

## Discussion

Previous studies have shown that BMPs can induce the formation of bone, dentin, cartilage, and connective tissues. BMP2 and 6 are considered to be more osteo-inductive than other BMPs (23–25). BMP9, also known as growth and differentiation factor 2, has previously been characterized as one of the most osteogenic inducers among BMP super-families (26,27). Our study explored the potential osteoblastic capabilities of BMP2, 6, and 9 on iPDBs by analyzing the mRNA expression of osteogenic genes and the formation of ectopic hard tissue. Because of the limited life span of normal PDBs and their time-consuming, laborious separation, we immortalized PDBs to facilitate the study of PDB biology in tooth engineering. We simplified recombinant adenovirus generation using the highly efficient Ad-Easy technology. Viral production is conveniently followed, and cells can be traced according to luminous intensity with the aid of GFP (13). Cre recombinase-mediated removal of SV40 T antigen decreases iPDB proliferation but partially mitigates the adverse impacts on iPDB multipotent differentiation potential (11). The endogenous expression results suggested that different expression levels of BMPs may partly explain differential osteogenesis. Kugimiya et al. (28) were the first to demonstrate that endogenous BMPs cooperatively play pivotal roles during *in vivo* bone formation under both physiological and pathological conditions. Each BMP may affect the mRNA expression of other BMPs in these cells.

In the present study, ALP activity increased significantly on the 5th and 7th days after BMP-9 overexpression, and matrix calcification nodule formation was observed 2 weeks after osteogenic induction. To better understand the effect of BMP9 on iPDB osteogenic differentiation, we analyzed the expression levels of bone-specific genes using semiquantitative RT-PCR. The osteogenic mRNA levels of OPN, OCN, BSP, MEPE, and DMP1 were upregulated by BMP9 stimulation relative to their levels in the GFP control group at the indicated time points, while SMAD6 was downregulated on days 5 and 7, with more significant downregulation occurring on day 7. These findings strongly suggest that BMP9 promoted osteogenic differentiation and mineralization throughout the entire osteogenesis process of PDBs; BMP9 regulated iPDB osteogenic differentiation more strongly than BMP2 or BMP6. In general, these differences were mainly due to their different osteogenic mechanisms. Nakashima et al. showed that BMP2/4 may induce tissue formation by triggering responsive cells to initiate signaling cascades (29,30), while Rutherford et al. found that BMP7 was not significantly mitogenic for the cells of the cell-rich layer. It could modulate their phenotypic expression and increase matrix formation (31,32). BMP6 has been shown to attract undifferentiated MSCs from the surrounding tissues and it can stimulate their proliferation and osteochondrogenic differentiation (33). The BMP9-mediated osteo-induction mechanism is completely different from those of other BMP family members, which determines its significant osteogenic ability to some extent. BMP9-induced osteogenic markers and matrix mineralization are not inhibited by noggin, an extracellular BMP antagonist, while noggin blunts BMP2, 4, 6, and 7-induced osteogenic markers and mineralization (34). Noggin inhibits TGF-β/BMP signaling by binding to BMPs with high affinity, therefore precluding their binding to specific cell surface receptors. BMPs can stimulate noggin production and each BMP has a different affinity for noggin. BMP7 has been shown to have a coordinated expression pattern during skeletogenesis (26,34).

The BMP canonical or SMAD-dependent signaling pathway is activated by the binding of a BMP to heterodimeric complexes of serine/threonine kinase receptors composed of type I and type II receptors (7,35). Upon binding of BMP to the receptor complex, SMAD1/5/8 phosphorylation occurs, resulting in the formation of heterodimeric complexes with SMAD4. The active complex is translocated to the nucleus and acts as a transcription factor, inducing the expression of BMP target genes (36). BMP9-induced nuclear translocation of SMAD1/5/8 is not inhibited by noggin, which strongly contributes to the potent osteogenic capability of BMP9 in MSCs (33,37). Furthermore, SMAD1/5/8-SMAD4 signaling can be blocked by SMAD6, which prevents the translocation of the heterodimeric complex to the nucleus (38). The decreased SMAD6 gene expression induced by BMP9 suggests that the osteogenic differentiation may be due, in part, to a suppression of BMP signaling by SMAD6. These results contribute to our understanding of the potent osteogenic capability of BMP9.

The ectopic experimental results further confirmed the osteogenesis superiority of BMP9 as follows: *in vivo*, mineralized masses were apparent at intramuscular injection sites in all groups, but masses at subcutaneous injection sites were only observed in the BMP9-induced group. Furthermore, compared to the masses in the other iPDBs, those in the BMP9-induced iPDBs exhibited greater mineralization and more orderly arrangement of bone-like trabeculae at the intramuscular injection sites. The iPDBs transfected with BMP6 exhibited greater mass volume and lower density than did those transfected with BMP2, but BMP2 induced more mature bone-like trabeculae and osteoid matrices than BMP6. These differences may have been partly due to noggin, which is a molecule that binds to BMP2 with high affinity but to BMP6 with low affinity (36,37). Furthermore, as the most active growth factor in osteogenesis known at present, BMP2 has high osteo-inductive activity and promotes bone repair in endochondral and membranous bones. Both Ad-BMP2 and 9 form bone through endochondral ossification after direct intramuscular injection, while Ad-BMP6 seems to induce bone by way of mechanisms similar to both direct intramembranous and indirect endochondral mechanisms (39).

Further research on the BMP receptors in iPDBs treated with BMPs and the specific osteogenic mechanisms is required to elucidate their different osteogenic activities.
